# Production performance of finisher broiler fed with cocoyam-corm meal as partial energy replacement for maize

**DOI:** 10.14202/vetworld.2016.1107-1112

**Published:** 2016-10-19

**Authors:** Christian Paul P. de la Cruz

**Affiliations:** Science Research Laboratory, College of Fisheries, Laguna State Polytechnic University – Los Baños Campus, Los Baños 4030, Laguna, Philippines

**Keywords:** broiler, cocoyam, feed-conversion ratio, Philippines, poultry, *Xanthosoma*

## Abstract

**Aim:**

The objective of this study was to evaluate the potential of Gabing San Fernando (*Xanthosoma* spp.) corms as partial carbohydrate replacement for maize in finisher broiler production.

**Materials and Methods:**

The completely randomized design was utilized to investigate the effects of three finisher poultry diets prepared in varying amounts of cocoyam-corm meal set at 0% (control), 25%, and 50% (experimental) replacement levels.

**Results:**

There were no significant differences (p≥0.05) as to mortality and body weight measurements between control and experimental groups. Similarly, the mean weights of selected internal organs and condemnable carcasses among treatment groups did not show any significant differences (p≥0.05). In terms of the average feed intakes, birds from 50%-cocoyam group had the highest mean value and were found to be statistically different (p≥0.01) from both control and 25%-cocoyam groups. However, feed conversion ratio did not significantly differ (p≥0.05) among three groups. Higher feed costs were associated with the 50%-cocoyam treatment diet, which was only consistent with higher feed inputs. Thus, the group fed with 50%-cocoyam meal had significantly higher total mean production costs (p<0.005) per bird, when other expenses were taken into account. The production costs for the group given 25%-cocoyam meal did not significantly differ (p≥0.05) from the control group.

**Conclusion:**

Partial replacement of maize with cocoyam-corm meal at 25% level was acceptable since inclusion at this level did not adversely affect the production performance of finisher broilers in terms of growth rate, mortality rate, and feeding efficiency. The use of cocoyam meal as nonconventional and alternative carbohydrate source in poultry diet presents positive economic implications, especially to smallhold farmers from the developing countries, like the Philippines.

## Introduction

Feeds constitute 60-80% of the total inputs in poultry production. High input costs associated with feed resources is considered a primary issue in animal production [1-3]. For instance, there is a scarcity of conventional feed ingredients like maize, which is considered the main source of energy for monogastric animals like chickens [4]. Insufficiency in the supply of maize poses problems since energy ingredients should account for 50-55% of the total formulated biomass of poultry feeds [5]. The competition among various consumers for maize that includes man, livestock, and fuel industries plus the high cost of this carbohydrate resource call for alternatives in poultry diets [6,7].

The utilization of relatively less expensive root and tubers as alternative sources of carbohydrate has been given attention in recent years. Studies have shown that with proper pre-processing techniques that eliminate antinutritional components, tuber meals such as cassava, sweet potato, and cocoyam are potential substitutes for maize meal [5]. The use of cocoyam meal as a partial replacement for maize has been investigated in various parts of Africa. The effects of graded levels of fermented wild cocoyam corm were investigated and were found that replacement set at 30% level was economical in broiler starter feeds [7]. Varying levels of sun-dried cocoyam corm meals as substitute ingredient in finisher broiler rations was found to have positive economic implications for diets with 15% cocoyam replacement [4]. Meanwhile, one study showed that cooked wild cocoyam meal set at 10-20% inclusion was better than its raw counterpart and comparable with control, i.e., full maize-based diet [8]. Another feeding trial done in Nigeria proved that boiled cocoyam meal at 50% inclusion level was comparable with control and even more economical, without adverse effect on growth parameters and feed efficiency of broilers [9].

There is still limited reference work on the utilization of cocoyam corms as alternative energy source in poultry production, especially in the Philippines. Thus, this study aimed to evaluate the potential of Gabing San Fernando (*Xanthosoma* spp.) corms as feed ingredient in finisher broiler rations. Specifically, this study determined and compared some important production parameters for finisher broilers fed with varying amounts of cocoyam meal as partial substitute for maize meal. The continued search for non-conventional carbohydrate resources that commands lower market price, locally available, less consumed by humans but could equally substitute maize in poultry rations is a matter of priority.

## Materials and Methods

### Ethical approval

This research was duly approved by the In-House Research Committee according to the LSPU Guidelines on Animal Research Ethics, for 40 days starting from 10^th^ November 2015 at the Poultry House of the University, with due care to minimize pain and discomfort to the birds.

### Research design

Three diet regimens modified at the level of cocoyam meal replacement set at 0% as control and 25-50% inclusion levels were developed and evaluated through a completely randomized design experimental design. The proportion of the feed ingredients was based on the standard basal diet for finisher broiler. Table-1 shows the feed composition of the three dietary treatments used in this study. All dietary treatments were submitted to the Feed Analysis Laboratory of the Animal and Dairy Science Cluster, College of Agriculture, UP Los Baños for proximate nutritional analysis that included moisture content, crude protein (CP), crude fiber (CFi), ether extract, and ash.

**Table-1:** Feed composition (in %) per kilogram of the three dietary treatments used as rations for finisher broilers.

Ingredient	Replacement level		

	0% CM	25% CM	50% CM
Maize meal	48	36	24
Cocoyam meal	0	12	24
Soybean meal	43	43	43
Rice bran	5	5	5
Limestone	2	2	2
Premix	0.5	0.5	0.5
Vegetable oil	1	1	1
Salt	0.5	0.5	0.5
Total	100	100	100

### Experimental procedures

A total of 45 broiler chicks from an established hatchery were used in this study. These were brought to the poultry house, acclimatized for one night with minimal feeding. Corms of Gabing San Fernando were obtained from the local public market. Other feed ingredients including maize, soybean, and premix were obtained from authorized dealers within Laguna. Corms were boiled for at least 1 h or until the skin can be easily peeled off. These were then set aside to cool for at least 30 min and subsequently shredded into smaller pieces. Air drying was allowed for at least 2 h, and subsequently, homogenized before mixing with feed ingredients. Broiler chicks were initially provided with chick booster for 10 days and starter feed for another 10 days. The feeds were manufactured by B-MEG. The treatment diets were introduced on day 21 until day 39. Experimental cages were thoroughly cleaned and dried before the chicks were housed. All materials necessary for brooding, feeding, and watering were made available to facilitate smooth operations. The chicks were reared for 20 days using commercial starter feed before the experimental diets were introduced for a 20-day feeding trial. All birds were culled at the end of the experiment and pertinent data were collected and analyzed.

### Data collection and analysis

The initial body weights of the birds, in grams, were determined before the application of the different dietary treatment. The body weights were monitored every 7 days. Digital weighing scale was utilized in determining the weights of the birds. Final live weights were determined at experimental termination. Organs and condemnable carcasses were consequently separated, dressing weight was determined, and feed efficiency parameters were subsequently computed. Production costs and corresponding returns were estimated for the three dietary regimens. All data were organized in SPSS version 20.0 statistical spreadsheet. Descriptive statistics were generated including mean values, mean differences, standard deviation, standard error of the mean, and 95% confidence interval. The univariate analysis of variance was employed to test the significance of the mean differences among treatments. Tukey’s HSD was also used for multiple comparisons. The tests were performed at 95% confidence level.

## Results

### Proximate nutritional constituents

Results of the proximate analysis on selected nutritional components indicated comparable scores for the three treatment diets (Table-2). Detailed inspection of the scores showed an increasing trend in terms of moisture content and nitrogen-free extract (NFE) with 50%-cocoyam diet having the highest moisture and NFE, whereas the control diet had the least. This result suggested that moisture content and NFE in feeds increases with increasing level of cocoyam inclusion. Decreasing patterns were observed for ash content, CFi, and crude fat (CF). In all parameters, the control diet showed the highest values, while the least values were observed in 50%-cocoyam diet. This trend indicates that ash content, CFi, and CF tend to decrease when the level of cocoyam meal replacement is increased. No apparent trend was observed for CP content among the three treatment diets; however, higher values were obtained in experimental diets as compared to the control diet. Thus, cocoyam-replaced meals appeared to have comparable proximate nutritional components when compared to a full-maize diet.

**Table-2:** Proximate nutritional constituents of the three dietary treatments in varying amounts of cocoyam meal as partial energy replacement for maize.

Constituents	Dietary treatment levels		

	0% CY meal	25% CY meal	50% CY meal
Moisture	10.0	11.6	12.6
Ash	7.34	6.56	6.76
CP	20.0	21.7	20.9
CFi	6.15	4.28	3.65
CF	13.6	12.4	11.9
NFE	42.9	43.5	44.2

CP=Crude protein, CFi=Crude fiber, CF=Crude fat, NFE=Nitrogen-free extracts,

### Growth rates and feed efficiency

Measures of mortality and growth rates were determined to reflect the performance of finisher broilers following a 20-day feeding trial (Table-3). In terms of live body weight measurements, higher mean values were noted for the control group as to final body weight, total weight gain, and average daily gain; whereas, higher dressing weights were measured in groups fed with experimental diets. As to the weight measures of condemnable carcasses, the higher values were recorded in the control group for both head and feet. However, the mean differences for all these parameters were not statistically significant (p≥0.05). These results suggested that the level of cocoyam replacement did not seem to affect the growth performance of the birds in terms of their body weights.

**Table-3:** Mean values (±SD) on growth rates of finisher broilers fed with varying amounts of cocoyam meal as partial carbohydrate replacement for maize.

Parameters	Replacement level				

	0% CM	25% CY meal	50% CY meal	p value	η^2^
Body weights					
IBW	671±32.5	671±31.6	721±39.0	0.202	0.41
FBW	1545±78.0	1525±44.0	1545±75.3	0.917	0.03
TWG	875±96.3	854±45.4	824.0±64.9	0.702	0.11
ADG	43.7±4.51	42.6±2.31	41.3±3.21	0.723	0.10
Dressing weight	1123±55.1	1142±34.0	1137±36.1	0.857	0.05
Condemnable carcass weights					
Head	50.0±12.2	46.0±4.18	46.0±8.94	0.730	0.05
Feet	82.0±8.37	77.0±6.71	70.0±7.07	0.072	0.36
Organ weights					
Liver/heart	52.0±14.4	40.0±0.00	54.0±5.48	0.059	0.38
Intestine	62.0±4.47	54.0±5.48	62.0±4.47	0.033	0.43
Gizzard	42.0±8.37	29.0±10.3	38.0±4.47	0.067	0.36
Mortality rate (%)	6.67±11.5	13.3±23.1	6.67±11.5	0.850	0.05

±=Standard deviation, η^2^=Effect size, ADG=Average daily gain, TWG=Total weight gain, FBW=Final body weight, IBW=Initial body weight

Measurements of the organ weights showed higher mean values for the gizzard in control group; whereas, the observed intestine and liver-heart weights were highest in the experimental groups. When statistical comparison was applied, a significant difference was not found among the three treatment groups in terms of their organ weights (p≥0.05), except for the intestine (p=0.033). However, the results of HSD *post-hoc* test did not classify the treatments into different subgroups (p≥0.05). These results indicated that there were comparable effects of the experimental diets on the organ weights of the birds, with the control diet. Significant differences (p≥0.05) were not also noted as to mortality rates among treatment groups, suggesting that inclusion of cocoyam meal in finisher broiler rations did not affect the general health and survival of the birds.

Results for the daily feed intake (DFI), total feed intake (TFI), and feed conversion ratio (FCR) are presented in Table-4. Regarding DFI of the birds, the control group showed the lowest mean value and then followed closely by the group in 25%-cocoyam diet. The highest DFI was observed in the group fed with 50%-cocoyam diet. The mean differences between the control group and 50%-cocoyam group were found to be statistically significant (p<0.005), indicating that birds fed with 50% cocoyam-substituted diets tended to consume more feed on a daily basis. Similarly, TFI was significantly lower in control group (p<0.005) and 25%-cocoyam (p<0.005); while 50%-cocoyam treatment group had higher TFI. The observed significant difference between control and 50%-cocoyam experimental group also indicate that partial inclusion of cocoyam meal at 50% inclusion level in finisher broiler diets apparently increases their feed intake. FCR was also computed among the three treatment groups to further examine the effects of partial cocoyam meal inclusion on the feed efficiency of finisher broilers. Results showed that the control group had the least, followed by 25%-cocoyam group and 50%-cocoyam group. Subsequent statistical testing revealed, however, that the mean differences for FCR among the three treatment groups were not significantly different (p≥0.05). It can be suggested that feeding finisher broilers with rations containing 25-50% cocoyam meal as partial substitute for maize would result to a comparable feeding efficiency observed in birds fed with full-maize ration.

**Table-4:** Mean values on feed efficiency of finisher broilers fed with varying amounts of cocoyam meal as partial carbohydrate replacement for maize.

Parameters	Replacement level				

	0% CM	25% CM	50% CM	p value	η^2^
DFI*	187.7±2.90^b^	189.5±1.80^b^	201.8±2.27^a^	0.001	0.91
TFI*	3566±55.0^b^	3600±34.2^b^	3833±43.1^a^	0.001	0.91
FCR	4.11±0.47^a^	4.22±0.19^a^	4.67±0.30^a^	0.148	0.47

* DFI and TFI are measured in grams, η^2^=Effect size,

a,b Mean values on the same row having the same superscripts are not statistically different. DFI=Daily feed intake, TFI=Total feed intake, FCR=Feed conversion ratio

### Production cost

Estimates of feed costs for the three treatment diets showed comparable values amounting to ₱30.00/kg. This was expected since the market price of maize and cocoyam corms were the same (₱30.00/kg) at the time of acquisition. However, the significant differences were noted on costs as related to the average feed intake of the birds (p<0.005). Higher feed costs were associated with the 50%-cocoyam treatment diet, corresponding to monetary values equal to ₱6.01 and ₱114.00 for DFI and TFI, respectively. The apparent increase in the feeding consumption of finisher broilers fed with 50%-cocoyam diet was consistent with higher feed inputs. Therefore, the experimental group reared in 50%-cocoyam diet had significantly higher total mean production costs (₱158.00; p<0.005) per bird, when all other expenses which include chick booster and grower feeds, vaccines, electricity, and labor were considered. Interestingly, the estimated production costs for the experimental group given 25%-cocoyam meal was not significantly different (p≥0.05) from the control group. The results presented above are summarized in Table-5.

**Table-5:** Comparative production costs associated with the three dietary treatments in varying amounts of cocoyam meal as partial carbohydrate replacement for maize.

Average measure	Replacement level			

	0% CM	25% CM	50% CM	p value
Cost of DFI per bird	₱5.55±0.09^b^	₱5.62±0.05^b^	₱6.01±0.07^a^	<0.005
Cost of TFI per bird	₱105±1.62^b^	₱107±1.02^b^	₱114±1.29^a^	<0.005
Total production cost per bird*	₱149±1.62^b^	₱151±1.02^b^	₱158±1.29^a^	<0.005
Returns per live weight sold**	₱20.6±7.30	₱17.0±4.33	₱11.7±7.11	0.300
Cost-benefit ratio***	0.14±0.05	0.11±0.03	0.07±0.04	0.242

a,b =Mean values on the same row having the same superscripts are not statistically different

* Total cost of treatment-feed given + ₱34.00 (pre-finishing feeds) + ₱10.00 (vaccines, labor, electricity, etc.).

** [Assuming market price of ₱110.00 per live weight sold×average final live weight (kg)] – total production cost.

*** Returns per total production cost/bird. DFI=Daily feed intake, TFI=Total feed intake

The economic value of cocoyam meal was further assessed by considering the return cost of investment per live weight sold. The market price of the average final live weight per treatment group was obtained by multiplying this value with the current market price of broiler per live weight sold, set at ₱110.00. Returns in peso were computed by deducting the total production costs from the market price according to the average dressed weight in kilogram.

Results showed no significant differences (p≥0.05) on the estimated profit margin. These were reflected in the obtained cost-benefit ratios that did not significantly differ (p≥0.05) among the three treatment groups. These results support the potential of cocoyam meal as economic feedstuff substitute for maize meal. Moreover, it should be emphasized that the cocoyam corms used in this study were food grade and due for human consumption, hence, commanded higher costs. The market price of cocoyam-based feeds is expected to reduce with growing supply of cocoyam corms for animal production purposes.

## Discussion

Many underexploited crops have been neglected despite the alarming global threats in food security. Tuber/root crops such as *Colocasia esculenta* and *Xanthosoma sagittifolium*, commonly called taro or cocoyam in several countries, are examples of underutilized crops that farmers and researchers continue to disregard. However, these crops show potentials to improve malnutrition, food scarcity [10], and production of farm animals that supply the majority of protein requirements among humans. It has been reported that cocoyam species produce corms that contain about 25% starch – in wet weight basis [11]; thus, making them good candidate sources of carbohydrate for poultry production [12,13]. To date, only a few studies have provided scientific evidence to support the idea for cocoyams as poultry feed ingredient. Therefore, this study was conducted to explore the possibility of using corms of *Xanthosoma* spp. as partial substitute for maize meal in finisher broiler rations.

Results of proximate analyses showed comparable values for CP, CFi, and ether extract. Similar findings were obtained in one study from Nigeria for CP of finisher broiler rations [9]. These findings are consistent with the results of the current study, whereby experimental feeds formulated with 25-50% cocoyam meal, in place of maize, had comparable nutritional components relative to a full-corn based control diet. This implies the suitability of cocoyam meal as feed ingredient in finisher broiler ration.

The production performance of some important poultry species fed with corm-replaced diets was also studied in different parts of the world. The effect on the growth performance and feed efficiency of quails (*Coturnix japonica*) fed graded levels of boiled sun-dried cocoyam (*C. esculenta*) was studied [14]. Replacement of maize with boiled cocoyam meal was found favorable for quails. The significant differences were not noted as to the growth performance and feeding efficiency between control group on 0% cocoyam-diet and experimental groups on 25% or 50% cocoyam-diets [14]. The use of cocoyam corms as partial feed ingredient in broiler ration was investigated in two studies done in Africa. The growth performance of finisher broilers fed with diets containing partial raw cocoyam corm meals in varying levels was investigated [4]. For another, the impact of raw and cooked wild cocoyam (*Caladium bicolor*) on the performance of broiler chicks was studied explored [8]; whereas, another study investigated the effects of sun-dried, soaked, and cooked/boiled *C. esculenta* on growth performance and carcass characteristics of broilers [15]. These studies demonstrated that partial replacement of maize with cocoyam-corm meal at 10-20% inclusion level did not show an adverse effect on growth performance of the birds. Moreover, it was also noted in both studies that the average estimated feed intake and FCR for experimental broilers fed with cooked-cocoyam diets did not significantly differ from the birds on the control diet. Boiling method seemed to reduce several antinutritional factors contained in cocoyam corms. These antinutritional factors include, among others, phytate, oxalate, tannin, saponin, and cyanide [13]. Thus, the use of boiled corms instead of raw was ideal in the partial application of cocoyam in broiler diets [16].

Similar findings were also noted in this study, in that, finisher broilers assigned to groups given feeds containing 25% and 50% boiled cocoyam corms had growth performance comparable with the control group. The results obtained in two studies involving broilers fed with diets containing *C. esculenta* [12] and *X. sagittifolium* [9] were also similar with the current study. On the other hand, conflicting results were obtained whereby the general health based on hematological analysis of the broilers was apparently normal as with the control, feeding them with the diets containing more than 10% cocoyam meal (*C. esculenta*) resulted in reduced growth rates [17].

In this study, increasing feed intake was noted with increasing inclusion level of cocoyam meal. Hence, experimental birds fed 50%-cocoyam diet showed the highest feed consumption; while those in control had the least. This finding was consistent with the results obtained in a previous study, and this was attributed to the higher metabolizable energy contained in corn relative to cocoyam [4]. It has been suggested that feeding efficiency of broilers is linked to metabolizable energy of the diet, in that, birds will tend to consume less of high energy feeds such as those containing full-maize component [1]. In contrast, the results obtained in two previous studies indicated that the feed intake was comparable between control and experimental group fed with boiled cocoyam [9,12]. The same findings were also reported in two independent findings [8,16]. Meanwhile, in all the studies mentioned above, as with the current findings, statistical comparisons of the FCR did not yield any significant differences. It can be noted, however, that the FCR in control group was consistently lower than the experimental groups. Therefore, the observed variability in the growth performances, feed intake, and feed conversion of broilers fed with cocoyam-based diets warrants further investigation. Subsequent reports should also indicate relevant statistical figures such as p-values and effect sizes to clearly define the effects of experimental treatments.

With regard to the analysis of production costs, increasing estimates were noted with increasing cocoyam-meal inclusion. This was consistent with the higher feed consumption rates observed in broilers given the cocoyam-based rations. Interestingly, the returns associated with experimental broilers were statistically comparable with the control. Similarly, the comparable production costs between control and those fed raw cocoyam-based diets were noted, and even reported better profit when boiled cocoyam corms were utilized [8,12]. The cost-effectiveness of feeding fermented cocoyam meal to laying quails was also explored and found that feeding the birds with 25%-cocoyam diet was economical [18].

## Conclusion

Results of this study demonstrated that cocoyam-corm meal could be used as partial substitute for maize at 25% inclusion level without adverse effects on the production performance of finisher broilers in terms of growth rate, mortality rate, feeding efficiency, and production cost. This study highlights the importance of neglected crops such as cocoyam in augmenting the pressure on maize as a major carbohydrate source in poultry diets. It also indicates the positive economic implications of using cocoyam meal in small-hold broiler production, especially in the developing countries.

## Authors’ Contributions

CPD conceptualized the study design, supervised the data gathering, and prepared and revised the manuscript prior to final approval for publication.
